# Perceived marginalization and mental health of young adults with migration backgrounds in South Korea: exploring moderating and mediating mechanisms

**DOI:** 10.3389/fpsyg.2023.1239337

**Published:** 2023-11-28

**Authors:** Sojung Jang, Jiwon Ryu, Kyu Jin Yon, Paul Youngbin Kim, Min Sun Kim

**Affiliations:** ^1^Department of Counseling and Educational Psychology, Indiana University Bloomington, Bloomington, IN, United States; ^2^Student Counseling Center, Sahmyook University, Seoul, Republic of Korea; ^3^Department of Psychology, Sogang University, Seoul, Republic of Korea; ^4^Department of Psychology, Seattle Pacific University, Seattle, WA, United States; ^5^Department of Psychology and Psychotherapy, Dankook University, Cheonan, Republic of Korea

**Keywords:** immigration, young adults, perceived marginalization, career maturity, mental health

## Abstract

This study explored the mediating effect of career maturity moderated by intimacy with parents and immigration backgrounds (native- or foreign-born young adults) on the relationship between perceived marginalization and the mental health of young adults with migration backgrounds (having mixed parentage of one Korean and one non-Korean immigrant parent) in South Korea. We collected data from 300 adults aged 25–34 with migration backgrounds (204 born in Korea and 96 born abroad) through the Gallup Research Institute of Korea and conducted a moderated-moderated mediation analysis using Model 21 of PROCESS Macro in SPSS. The analysis showed that career maturity moderated by intimacy with parents and migration backgrounds mediated the relationship between perceived marginalization and mental health. However, the results were only significant for participants who were born abroad and immigrated to Korea, and not for those who were born in Korea. These findings suggest that while greater perceived marginalization leads to lower career maturity and negatively impacts the mental health of foreign-born young adults, higher levels of intimacy with parents can buffer these negative effects.

## Introduction

Globalization in the 1900s led to a considerable increase in international marriages and job opportunities worldwide, which in turn triggered the rapid growth of families with a new type of ethnic diversity, referred to as *multicultural families* (Kim and Okazaki, 2022). This growth occurred even in ethnically homogenous nations such as South Korea (hereafter Korea) (Jeong, 2009). Despite the growth of such families, Korea’s deeply rooted ethnic homogeneity and nationalism might mean that Koreans’ perspectives regarding multiculturalism differs from those of other multiethnic countries. For example, the strong one-ness of Koreans carries critical historical meaning, as it has united the entire country to collectively resist past foreign invasions and wars. Korea’s cultural and historical background has also created a Korean mono-ethnicity that limits the country from fully embracing global values; put differently, Korean society features a superficial form of multiculturalism (Lee, 2012; Kim et al., 2019). Therefore, immigrants to Korea and children of transnational marriage families are often treated as foreigners, making them vulnerable to various stereotypes and discriminatory acts (Yoo and Kim, 2015), which can lead to marginalization and mental health problems. While researchers have recognized the aforementioned challenges among children with migration backgrounds (e.g., Ha and Kim, 2013; Lim, 2013), they have tended to focus on adolescents. Even the Korean Multicultural Family Support Act only covers immigrant children under 24, leaving the impacts of marginalization among young adults with migration backgrounds (hereafter YAMB) unexplored.

The current study attempted to fill this gap by investigating the relationship between perceived marginalization and mental health among young adults with migration backgrounds (having mixed parentage of one Korean and one non-Korean immigrant parent) and exploring the mediating effect of career maturity along with the moderating roles of intimacy with parents and immigration backgrounds (native-born or foreign-born young adults). This study was partially guided by the *Systems Theory Framework of Career Development* (STF; McMahon and Patton, 1995), which emphasizes the contexts of individuals’ career decision-making and career transitions. According to STF, contextual factors including socioeconomic status and globalization can affect individuals’ careers, which, in turn, can affect their mental health both positively and negatively (e.g., stress and self-worth; Hulin, 2002; McMahon, 2017, 2021).

**Figure 1:**
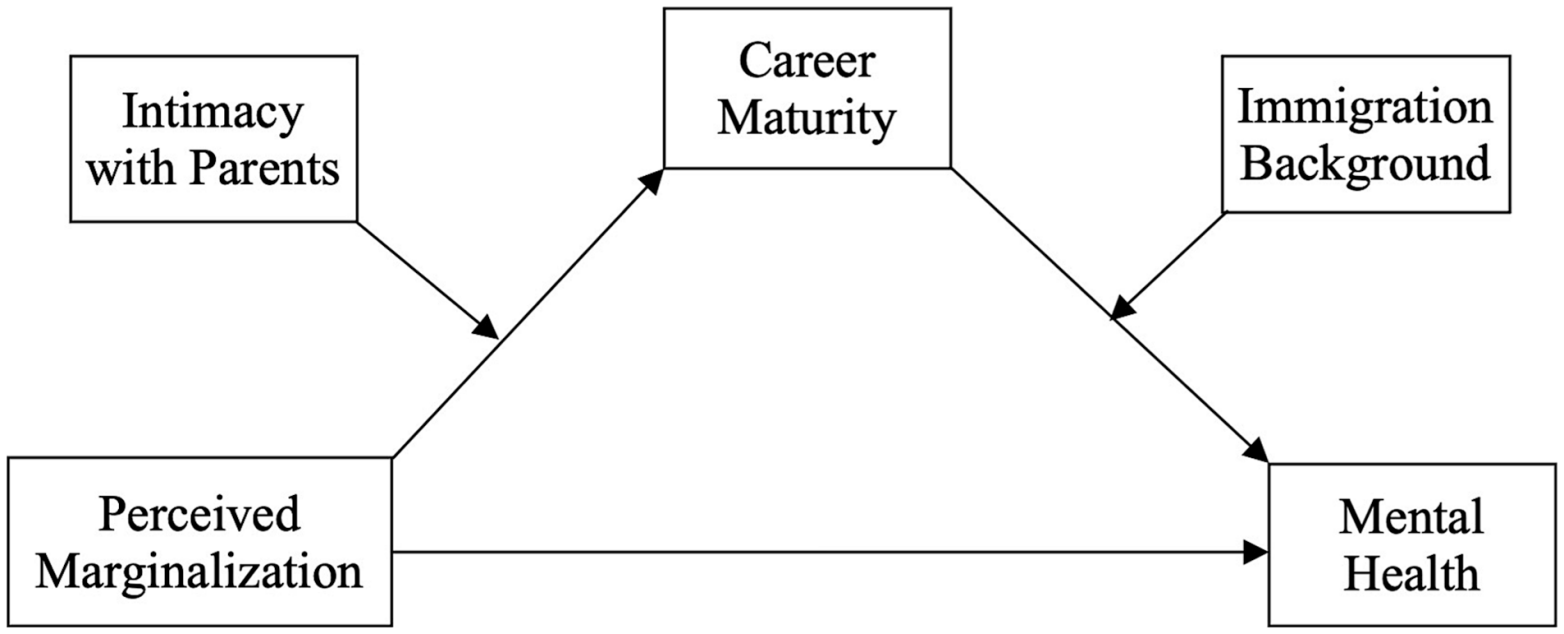
This figure displays the moderated-moderated mediation model showing the mediation effect of career maturity moderated by intimacy with parents and immigration background on the relationship between perceived marginalization and mental health.

Specifically, considering the contextual elements of YAMB’s experiences, we examined whether *perceived marginalization* (Issmer and Wagner, 2015), defined as the subjective feeling that one is socially excluded due to one’s low social status or importance, is associated with the mental health of YAMB. In addition, we explored the mediating effect of *career maturity*, defined as one’s readiness to cope with career developmental tasks (Super, 1990), on the relationship between perceived marginalization and mental health. In young adulthood, selecting and initiating a vocation is crucial to the development of one’s identity (Winderman et al., 2018) and well-being (Lent and Brown, 2008), while perceived social ostracization can hinder one’s career maturity (Crites, 1973; Autin et al., 2017) and negatively affect mental health (Lee, 2004; Lee et al., 2008). To gauge the effects of social support, we also explored the moderating effect of *intimacy with parents* on this mediation mechanism. Lastly, to develop a nuanced understanding of different immigrant backgrounds, we explored the difference in the aforementioned mediating mechanism between Korean-born YAMB and foreign-born YAMB. Recognizing that the simultaneous challenges of adapting to life in Korea and transitioning into adulthood require foreign-born YAMB to perform distinct developmental tasks, we attempted to explore whether immigration background (native-born vs. foreign-born) moderates the mediating effect of career maturity on perceived marginalization and mental health. Figure 1 displays the study’s model, and the rationale is outlined below.

## Perceived marginalization and mental health

In addition to overt discrimination, marginalized or underserved populations can experience difficulties stemming from the perception that their affiliate groups have lower social positions with less power (Kim and Baek, 2021). Marginalization theory posits that the more one is underprivileged (e.g., those with a migration background, cultural, ethnic, and racial minorities), the more one is aware of society’s unfair gaze or treatment (Crocker et al., 1998; Issmer and Wagner, 2015). Moreover, perceived marginalization can be shaped by contextual factors, such as the negative generalizations about one’s own group; in a recent study, U.S. Latinx individuals who were citizens or permanent residents still demonstrated an empirical association between the fear of others questioning their legal status in their own land (“perceived illegality”) and mental health distress, attesting to the notion that perceived marginalization, regardless of competing realities (e.g., legal reality that says it is highly unlikely to be deported), can still have a notable impact on mental health distress (Santos et al., 2021). These experiences negatively affect their views of the world (Sigelman and Tuch, 1997; Vorauer et al., 2000), as marginalized individuals attribute the discrimination that they experience to the larger society; this can eventually develop into negative meta stereotypes (i.e., the perception that society will always harbor prejudice and negative stereotypes against them and their groups, Sigelman and Tuch, 1997; Owuamalam and Zagefka, 2011).

In addition, researchers have found that as perceived marginalization and negative meta stereotypes increase, people feel more powerlessness because they lack the ability to control and choose their goals; the inability to find purpose in one’s life fosters feelings of meaninglessness, harming one’s mental health (Dean, 1961; Calabrese and Adams, 1990; Mau, 1992; Issmer and Wagner, 2015). The subjective feeling that one’s group is relatively unimportant and receives less social recognition in the areas of economics, culture, and politics negatively affects the overall career development and mental health of marginalized individuals (Dwivedi et al., 2007; Thomas and González-Prendes, 2009; Autin et al., 2017; Baah et al., 2019). As an example, the South Korean government’s disaster relief fund policy during the COVID-19 pandemic unfairly discriminated against immigrants by excluding them from the list of beneficiaries (Lee et al., 2021). Such experiences can lead one to feel a sense of feeling little control or agency over life circumstances. Those who are experiencing social marginalization or oppression can sometimes resort to a worldview emphasizing the individual’s powerlessness over their lives, and internalizing a view emphasizing that control resides outside of oneself (Sue, 1978). These negative impacts of social marginalization and ostracization are not confined to certain developmental periods; in fact, they can intensify over time (Aya and Kudo, 2010; Bäckman and Nilsson, 2011). Thus, exploring whether perceived marginalization is associated with mental health among YAMB in Korea is important.

## Perceived marginalization and career maturity

Social standing is often associated with career maturity. Previous studies (Diemer and Blustein, 2007; Soresi et al., 2012) have shown that individuals from more privileged social backgrounds with fewer experiences of marginalization perceive less internal and external barriers to their vocational success, enabling them to more easily develop career maturity. On the other hand, young adults who perceive themselves as marginalized are likely to experience psychosocial stressors, such as discrimination and socioeconomic inequity (Yoo and Kim, 2015), which can lead to lower self-esteem, less self-control, and other traits related to career immaturity (Duffy et al., 2016). Owuamalam and Zagefka (2011) found that viewing one’s own group as socially inferior and therefore prone to discrimination can trigger a sense of inferiority and limit the effort one devotes to moving up the social ladder via career exploration and job search, negatively affecting career development.

Moreover, studies have found that perceived marginalization limits one’s expectations regarding the achievements of one’s own group, which can constrain work volition (Diemer and Blustein, 2007; Autin et al., 2017) and adaptive career attitude development (Douglass et al., 2020), ultimately adversely affecting one’s career maturity. Studies conducted in Korea have revealed that multicultural adolescents experience more hardships in overall career development than other adolescents (Jung and Kim, 2020; Kim J. E., 2020; Kim H. S., 2020; Yoon, 2020), including intimidation, unwillingness to engage in career exploration, and difficulties in career decision-making processes (Lee et al., 2019). Based on these studies, we predicted that the perceived marginalization of YAMB would be negatively associated with their career maturity.

## Career maturity and mental health

A career is an essential aspect of early adulthood that meaningfully affects identity development and overall life satisfaction (Winderman et al., 2018). Researchers have found that doing what one likes increases one’s sense of accomplishment, satisfaction, and well-being (Jadidian and Duffy, 2012; Duffy et al., 2016). Related, a study with Korean middle school students revealed that following participation in a short-term career exploration program, they demonstrated more career maturity, satisfaction with school, and well-being; moreover, the same study highlighted the overall benefits of career maturity boosted by engagement in career exploration, as the pre-program career maturity of participants was associated with their post-program school satisfaction (Ham and Lim, 2017). Moreover, studies have shown that career and employment-related stress is associated with negative physiological symptoms (e.g., headache and stomachache), behavioral symptoms (e.g., crying, criticism, and eating disorders), and emotional symptoms (e.g., depression, agitation, frustration, helplessness, suicidality) (Lee, 2004; Lee et al., 2008). Lee et al. (2000) reported that among Koreans in their 20s, those experiencing employment failure reported the lowest mental health and life satisfaction levels. Alongside job stress and employment failure, career indecision can lead to negative mental health outcomes such as decreased well-being and increased distress (Winderman et al., 2018), as well as diminished academic performance, which can also lead to stress (Park et al., 2008).

Given that job satisfaction serves as a significant gauge of the well-being of immigrants (Wang and Jing, 2018), identifying a suitable career and securing a relevant job can be an important correlate of life satisfaction among YAMB. The YAMB who are underprepared to find careers that align with their abilities, interests, personality characteristics, and values due to perceived marginalization can show lower career maturity and experience mental health problems (Super, 1953; Duffy et al., 2016). Therefore, based on previous research, we predicted that perceived marginalization would negatively affect YAMB’s mental health through career maturity.

## Intimacy with parents

Social support from family, friends, and communities can serve as a positive contextual influence that helps individuals overcome the stress triggered by experiences of marginalization or ostracization. It can help individuals challenge the legitimacy of the discrimination they face and mitigate the negative thoughts they have about themselves (Pascoe and Richman, 2009) by buffering the impacts of racial, gender, or socioeconomic stresses on their mental health (Duffy et al., 2016). As one of several potential sources of social support, parents can play an especially vital role, specifically for YAMB, as parental support helps academic performance, career development, psychological stability, and overall adaptation (Obradović et al., 2013; Lee and Kim, 2016). Especially in contexts that clearly differentiate between in-group and out-group relationships (e.g., see Markus and Kitayama, 1991) and also emphasize the importance of respect for social hierarchy (e.g., vertical collectivism; Singelis et al., 1995), the examination of parental influence as a unique and relevant source of support is warranted.

Therefore, we expected that even when the perceived marginalization experienced by YAMB negatively affects their career maturity and mental health (Pascoe and Richman, 2009), high levels of intimacy with parents would buffer this empirical relationship. Thus, we hypothesized that intimacy with parents would moderate the mediating effect of career maturity on the relationship between perceived marginalization and mental health.

## Immigration status (native-born and foreign-born YAMB)

YAMB who were born abroad and have immigrated to Korea are exposed to stressors beyond those experienced by multicultural young adults born in Korea, including cultural adaptation, racial discrimination, economic problems, social relationship, and acculturative stress (Park, 2010; Ha and Kim, 2013; Yoo and Kim, 2015; Kim, 2017). For example, adolescents with migration backgrounds in Korea (i.e., not born in Korea) experience unexpected gaps in their schooling (e.g., having to take time off from school while waiting for visas) or uncertainty about visa status due to immigration, which can exacerbate their identity confusion, self-deprecation, and lack of self-esteem. Troublingly, a recent study in Korea demonstrated that 80% of these adolescents above the age 14 were not attending school (Kim, 2020b). Furthermore, they reported considering suspending their studies 1.7 times more than those born in Korea, and those who had already left school reported a lack of career identity (Lee, 2016). Research has also shown that immigrant youths, even those who have not attended school in Korea, are also exposed to stress during migration due to not seeing their parents as often (e.g., due to having to adapt to a new environment), racial discrimination, cultural and language barriers, and higher career expectations stemming from a desire for greater achievement while migrating to Korea (Bai, 2017). Since the challenges facing adolescents can continue to exert influence into early adulthood, we expected that the impacts of marginalization on career maturity and mental health would be more pronounced among young adults who immigrated to Korea than their Korean-born YAMB counterparts. Therefore, this study hypothesized that individuals’ immigration backgrounds (native-born vs. foreign-born) would moderate the mediating effects of career maturity on the relationship between perceived marginalization and mental health.

## Method

### Participants and procedure

Three hundred immigrants in their early adulthood living in South Korea were recruited to participate in this study. We collected data with the assistance of Korea Gallup Research Institute (KGRI). KGRI recruited participants through multicultural organizations such as the multicultural family support center located in various regions of Korea. Recruiters from KGRI conducted one-on-one interviews with participants to provide them a general introduction to our survey and obtain their informed consent and responses to the survey. The survey was approved by the Institutional Review Board of XXX University in South Korea (IRB ###).

We defined our sample of “young adults with migration backgrounds (YAMB)” as those aged 25 to 34 with one Korean parent and one non-Korean immigrant parent. Among these individuals, we further categorized the sample into two groups: one group consisted of 204 Korean-born young adults with one immigrant parent and one Korean parent (referred to as “native-born YAMB” in this study); the other consisted of 96 young adults who were born abroad and migrated to Korea, with one immigrant parent and one Korean parent (referred to as “foreign-born YAMB” in this study).

Recognizing that many studies of multiculturalism have focused on younger cohorts (with target populations typically between middle school and college students), we intentionally recruited those between 25 and 34 years of age, an economically active age group within the 15–34 young adult category specified by the South Korean Youth Framework Act (Kim, 2021). Among the participants, 125 were male (41.7%), and 175 were female (58.3%), with an average age of 28.87 (*SD* = 2.76). One hundred and forty-five participants resided in Gyeonggi (48.3%), 33 in Seoul (11%), 26 in Busan (8.7%), 17 in Incheon and Daejeon (5.7%, respectively), 11 in Gangwon, Chungnam, and Gyeongnam (3.7%, respectively), 6 in Chungbuk (2%), 4 in Jeonbuk, Jeonnam, and Gyeongbuk (1.3%, respectively), 4 in Daegu (1.3%), 3 in Jeju (1%), and 2 in Gwangju and Ulsan (0.7% each).

The majority of participants reported their socioeconomic status as “middle” (54.7%), followed by “low to middle” (31%), “middle to high” (8.6%), “low” (4.7%), and high (1%). Lastly, the highest level of education reported by the participants was “non-high school graduate” (2, 0.7%), “high school graduate” (72, 24%), “certificate or enrollment in college” (32, 10.7%), “certificate or enrollment in university” (52, 17.3%), “certificate or enrollment in graduate program” (5, 1.7%), “college graduate” (55, 18.3%), and “university graduate” (82, 27.3%), with a majority having acquired a certificate, enrollment, or graduation from university.

## Measures

### Perceived marginalization scale (Gruppenbezogene Menschenfeindlichkeit: GMF)

We assessed perceived marginalization using a four-item scale that Issmer and Wagner (2015) developed using items from the German GMF survey (Gruppenbezogene Menschenfeindlichkeit; see Heitmeyer and Mansel, 2008). We used the Korean version translated by Kim and Baek (2021). The GMF measures the respondents’ perceived social position or status as a member of a group. Sample items include: “People like me are worth less than others in Korean society” and “In our society, people like me are not offered any chances.” Kim and Baek (2021) made only one minor modification to the items (modifying “German society” to “Korean society”). Participants rated each item on a scale from 1 (*strongly disagree*) to 4 (*strongly agree*), with a higher score indicating higher perceived marginalization. The Cronbach’s α of the perceived marginalization scale in Kim and Baek’s (2021) study was 0.83, and the Cronbach’s α in our study was 0.84.

### Career maturity scale

We used the Career Maturity Scale developed by the National Youth Policy Institute (National Youth Policy Institute, 2010). The scale consists of seven items designed to reflect factors in Crites and Savickas’ (1978) career maturity model, such as competence and attitudes toward career decision-making. Sample items include: “I am still unaware of my aptitude or talent,” and “It is pointless to choose a specific vocation at this stage because the future is filled with uncertainty anyway.” Participants rated each item on a 5-point Likert scale ranging from 1 (*strongly disagree*) to 5 (*strongly agree*), and we reverse coded responses so that higher scores indicated higher career maturity. While the Korean Youth Panel Survey (KYPS) showed varying Cronbach’s αs from 0.55 to 0.78 depending on each measurement, the Cronbach’s α in our study was 0.81.

### General health questionnaire

To measure the mental health of young adults with migration backgrounds, we used a 12-item general health scale that abbreviates the original 60-items scale developed by Goldberg and Hillier (1979). GHQ-12 is designed to determine the current psychological state of respondents by asking how their mood has changed relative to their normal state over the preceding two to three weeks. We used the Korean version of GHQ-12 translated and validated by Park et al. (2012). The sample items include: “I can focus on what I am doing” and “I feel overwhelmed by the problems I face.” Participants responded to items on a four-point Likert scale ranging from 1 (*always*) to 4 (*never*). We reverse-coded positive questions so that higher scores indicated more mental health problems. The Cronbach’s αs of the scale were 0.88 in Park et al. (2012) and 0.71 in our study.

### Parental intimacy scale

We used items from the 4th Family Factual Survey conducted by the South Korean Ministry of Gender Equality and Family (2020) to measure intimacy with parents. Questions were designed to measure participants’ overall satisfaction and relationship quality with their parents. Sample items include: “I can talk to my parents about my worries and concerns” and “I feel intimate with my parents.” We removed one item (“My parents do not understand me very well”) due to low reliability. Participants responded to items on a five-point Likert scale ranging from 1 (*never*) to 5 (*always*), with higher scores indicating more satisfaction with their relationships with their parents. The Cronbach’s α of the scale was 0.77 in this study.

### Demographic information

We controlled age, gender, immigration status, place of residence, and perceived socioeconomic status in our analysis.

### Data analysis plan

We analyzed the data using SPSS 25.0 and the PROCESS Macro for SPSS 2.16 (Hayes, 2013). PROCESS is a convenient tool that is widely used to examine models containing one or more moderating and mediating variables as well as covariates (Hayes, 2013). First, we conducted a Pearson correlation analysis to examine the bivariate correlations between the variables. Then, we used Models 4 and 21 of the PROCESS macro to conduct the mediation and moderated-moderated mediation analyses while controlling for age, gender, and socioeconomic status. In our mediation and moderated mediation analyses, we utilized bootstrapping procedures with 5,000 bootstrap samples and a 95% confidence interval.

## Results

### Preliminary analysis

Table 1 displays the means, standard deviations, and skewness and kurtosis values of the study’s variables. Our analysis revealed that the variables’ absolute values of skewness and kurtosis did not exceed 2 and 7, respectively, satisfying the normality assumption (Curran et al., 1996).

**Table 1:** Description of study variables and correlations (*N* = 300).

		1	2	3	4	5	6	7	8
1	Mental Health	1							
2	Social Marginalization	0.457^**^	1						
3	Career Maturity	−0.263^**^	−0.281^**^	1					
4	Intimacy with Parents	−0.304^**^	−0.368^**^	0.159^**^	1				
5	Immigration Background	−0.015	0.187^**^	−0.056	−0.107	1			
6	Age	0.022	0.066	0.130^*^	−0.115^*^	0.102	1		
7	Socioeconomic Status	−0.178^**^	−0.127^*^	0.121^*^	0.161^**^	0.024	0.064	1	
8	Gender	0.119^*^	0.048	−0.010	0.055	0.043	−0.007	0.036	1
	Average	1.89	1.64	3.70	3.69	0.32	28.87	2.7	0.58
	Standard Deviation	0.35	0.59	0.62	0.60	0.47	2.76	0.73	0.49
	Skewness	0.44	0.52	−0.17	−0.22	0.78	0.18	−0.04	−0.34
	Kurtosis	−0.14	−0.79	−0.49	−0.15	−1.41	−1.19	0.50	−1.90

**p < 0.01, *p < 0.05.

Immigration status was dummy coded with the native-born YAMB as 0 and foreign-born YAMB as 1. In terms of gender, made was coded as 0 and female was coded as 1.

To check for multicollinearity (Table 1), we examined the correlations between the study variables. Because the absolute values of the correlation coefficients were all less than 0.5, multicollinearity was not a concern (Kline, 2005). Our analysis revealed significant correlations between social marginalization and career maturity (*r* = −0.281, *p* < 0.01), mental health (*r* = 0.457, *p* < 0.01), and intimacy with parents (*r* = −0.304, *p* < 0.01). Likewise, we found significant correlations between career maturity and mental health (*r* = −0.263, *p* < 0.01) and intimacy with parents (*r* = 0.159, *p* < 0.01), as well as a significant correlation between mental health and intimacy with parents (*r* = −0.304, *p* < 0.01). Finally, the analysis showed a significant correlation between migration background (dummy coded: native-born YAMB as 0, and foreign-born YAMB as 1) and social marginalization; specifically, we found that foreign-born YAMB perceived themselves as more social marginalized (*r* = 0.187, *p* < 0.01).

### The mediating effect of career maturity on the relationship between perceived marginalization and mental health

To examine the mediating effect of career maturity on the relationship between perceived marginalization and mental health, we conducted a simple mediation analysis using Model 4 of the PROCESS Macro for SPSS. We controlled for age, gender, and socioeconomic status to account for the impacts of personal context on mental health (Garcini et al., 2016). The results showed that the total effect of perceived marginalization on mental health was significant (*β* = 0.26, *t* = 8.46, *p* = 0.000). Also, the direct positive effect of the perceived marginalization on mental health (*β* = 0.23, *t* = 7.47, *p* = 0.000), the negative effect of perceived marginalization on career maturity (predictor to mediator; *β* = −0.30, *t* = −5.04, *p* = 0.000), and the negative effect of career maturity on mental health (mediator to outcome; *β* = −0.08, *t* = −2.60, *p* = 0.0098) were all statistically significant. Results based on 5,000 bootstrapped samples revealed a statistically significant indirect effect of 0.02, with the lower limit of the confidence interval at 0.0057 and the upper limit at 0.0440 (see Table 2). These results indicate that the individuals who experienced greater perceived marginalization were more likely to suffer a stronger negative effect on their mental health. At the same time, we also found that greater perceived marginalization was related to lower career maturity, which was associated with higher negativity in mental health. This finding suggests that perceived marginalization is related to mental health through career maturity.

**Table 2:** Mediation effect of career maturity on the relationship between perceived marginalization and mental health.

	Predictor	Dependent variable	*ß*	*SE*	*T*
	Perceived marginalization	Career maturity	−0.30	0.06	$-5.04^{***}$
	Career maturity	Mental health	−0.08	0.03	$2.60^{**}$
	Perceived marginalization	Mental health	0.23	0.03	$7.47^{***}$
	ß	SE	T	LLCI	ULCI
Total effect	0.26	0.03	$8.46^{***}$	0.20	0.32
Direct effect	0.23	0.03	$7.47^{***}$	0.17	0.29
	Boot indirect effect	Boot SE	Boot LLCI	Boot ULCI	
Indirect effect	0.02	0.01	0.01	0.04	

***p < 0.001, **p < 0.01.

### Intimacy with parents and immigration background as moderators

To examine whether intimacy with parents and participants’ immigration backgrounds moderated the mediating effect of career maturity on the relationship between perceived marginalization and mental health, we conducted a moderated-moderated mediation analysis using Model 21 of the PROCESS Macro for SPSS (refer to Figure 1). In all analyses, we controlled the effects of age, gender, and socioeconomic status. Table 3 displays the results.

**Table 3:** Mediation effect of career maturity moderated by intimacy with parents and immigration status on the relationship between perceived marginalization and mental health.

			ß	SE	LLCI	ULCI
Perceived marginalization → Career Maturity			−1.36	0.36	−2.05	−0.66
Intimacy with Parents → Career Maturity			−0.42	0.17	−0.76	0.08
Perceived marginalization × Intimacy with Parents → Career Maturity			0.30	0.10	0.11	0.49
Conditional indirect effect based on the differences in with-parent intimacy	Low (Average-1SD)		−0.45	0.08	−0.61	−0.28
	Medium (Average)		−0.25	0.06	−0.37	−0.12
	High (Average + 1SD)		−0.04	0.10	−0.23	0.14
Career Maturity → Mental health			−0.03	0.04	−0.10	0.04
Immigration Status → Mental health			0.40	0.22	−0.03	0.82
Career Maturity × immigration Status → Mental health			−0.13	0.06	−0.24	−0.01
Conditional indirect effect based on the differences of immigration status	Native-born		−0.03	0.03	−0.1	0.3
	Foreign-born and immigrated		−0.16	0.04	−0.26	−0.07
Moderated-moderated mediation			−0.04	0.02	−0.09	−0.003
Conditional indirect effect based on the differences in intimacy with parents and immigration status	Low intimacy with parents	Native-born	0.01	0.02	−0.02	0.05
	Low intimacy with parents	Foreign-born and immigrated	0.07	0.03	0.03	0.13
	Medium intimacy with parents	Native-born	0.01	0.01	−0.01	0.03
	Medium intimacy with parents	Foreign-born and immigrated	0.04	0.02	0.01	0.07
	High intimacy with parents	Native-born	0.001	0.005	−0.01	0.01
	High intimacy with parents	Foreign-born and immigrated	0.01	0.02	−0.03	0.04

First, we found that intimacy with parents and migration backgrounds had significant moderating effects on the relationship between perceived marginalization and career maturity. As noted earlier, the higher the perceived marginalization, the more likely participants were to suffer lower career maturity. However, the path from perceived marginalization to career maturity was only significant when levels of intimacy with parents were low (*β* = −0.45, BC 95% CL [LLCI = −0.61, ULCI = −0.28]) or moderate (*β* = −0.25, BC 95% CL [LLCI = −0.37, ULCI = −0.12]) but not when the level of intimacy with parents was high (*β* = −0.04, BC 95% CL [LLCI = −0.23, ULCI = 0.14]).

**Figure 2:**
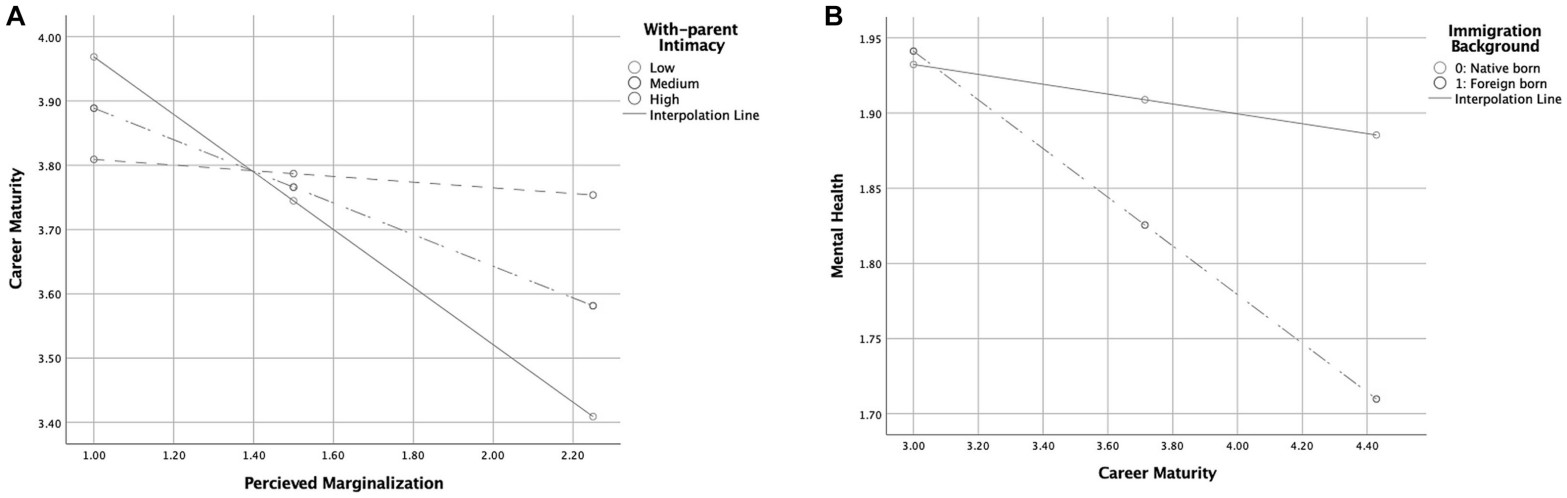
This figure demonstrates **(A)** the moderating effect of intimacy with parents on the relationship between perceived marginalization and career maturity, and **(B)** the moderating effect of immigration status on the relationship between career maturity and mental health.

Meanwhile, although we found a significant association between career maturity and mental health among foreign-born YAMB (*β* = −0.16, BC 95% CL [LLCI = −0.26, ULCI = −0.07]), we did not find such an association among native-born YAMB (*β* = −0.03, BC 95% CL [LLCI = −0.10, ULCI = 0.04]). Figures 1, 2 display the moderating effects.

We also examined the moderated-moderated mediation index as suggested by Hayes (2013) to determine the significance of the moderated-moderated mediation effect. The index was −0.04, and the bootstrap confidence interval did not include a 0 (BC 95% CL [LLCI = −0.09, ULCI = −0.003]), indicating that the moderated mediation effect was statistically significant. To further investigate the moderated-moderated mediation effect, we also examined the indirect conditional effect, testing the indirect effect of perceived marginalization on mental health through career maturity based on differences in levels of intimacy with parents and immigration backgrounds. Our results indicated that while intimacy with parents did not significantly moderate the indirect relationship between perceived marginalization and mental health through career maturity among native-born YAMB (*β* = −0.01, BC 95% CL [LLCI = −0.04, ULCI = 0.01]), the moderation was significant for foreign-born YAMB (*β* = −0.05, BC 95% CL [LLCI = −0.10, ULCI = −0.01]); that is, intimacy with parents moderated the indirect relationship between perceived marginalization and mental health through career maturity among the foreign-born YAMB. More specifically, the indirect effect of perceived marginalization in predicting mental health through career maturity was significant when levels of parental intimacy were low (average − 1SD, BC 95% CL [LLCI = 0.03, ULCI = 0.13]) or moderate (average, BC 95% CL [LLCI = 0.01, ULCI = 0.07]) but not when the level of parental intimacy was high (average + 1SD, BC 95%, CL [LLCI = −0.03, ULCI = 0.04]).

In sum, career maturity did not have a significant mediating effect on the relationship between perceived marginalization and mental health regardless of the level of intimacy with parents among native-born YAMB. However, the mediating effect was significant among foreign-born YAMB when their levels of intimacy with their parents were low or moderate.

## Discussion

This study explored (a) the mediating effect of career maturity on the relationship between perceived marginalization and mental health, and (b) the moderating effects of intimacy with parents and immigration background in the mediation model (perceived marginalization → career maturity → mental health) among 300 young adults with migration backgrounds (YAMB) living in Korea. We found an association between perceived marginalization and mental health through career maturity as the mediator. Also, the moderated-moderated mediation analysis indicated that the impact of perceived marginalization on mental health through career maturity was moderated by intimacy with parents and immigration background. We found that a high level of intimacy with parents mitigated the adverse effect of social marginalization on mental health through career maturity, but this finding only manifested among foreign-born YAMB, not native-born YAMB. Below, we discuss each finding in detail.

First, our analysis identified career maturity as a significant mediator in the relationship between perceived marginalization and mental health. Specifically, it showed that both the direct effect of perceived marginalization on mental health and its indirect effect through career maturity were significant. Previous studies have found that high levels of perceived discrimination among marginalized individuals can unfavorably impact career development indicators such as career maturity, work volition, career adjustment, and career satisfaction (Savickas, 2002; Duffy and Dik, 2009; Duffy et al., 2012). In particular, when individuals from underrepresented groups experience lower levels of career maturity combined with a lack of work volition or career adaptability, they tend to experience difficulty in career decision-making and transitioning to jobs, which in turn might prevent them from finding satisfaction in their careers (Jeong and Cho, 2012). Because work fulfills fundamental human needs, such as the need for self-determination (Blustein, 2013), failure to find a satisfactory job and dissatisfaction with career decisions can lead to negative mental health outcomes (Choi and Kim, 2007). Prior studies (e.g., Axelsson and Ejlertsson, 2002) have demonstrated that young adults experiencing unemployment issues were more likely to experience mental health problems such as depression, anxiety, and insomnia; similarly, research has shown that Korean college students in their final year of college who went through prolonged periods of not being able to find satisfactory jobs also experienced mental health distress (Chang et al., 2004). Taken together, the findings of these studies align with the finding that a higher level of perceived social marginalization among YAMB is negatively associated with their career maturity, which in turn leads to more mental health difficulties.

Second, we found that career maturity moderated by intimacy with parents and immigration background had a significant mediation effect on the relationship between perceived marginalization and mental health. More specifically, the results suggested that perceived social marginalization among YAMB was negatively associated with their career maturity, but intimacy with parents helped buffer this negative association (see Figure 2A). This finding aligns with previous research showing that healthy interpersonal relationships play a buffering role in stressful situations (Cohen and Wills, 1985) as well as helping individuals with their career decisions and overall career development processes (Blustein, 2011). In addition, emotional support from positive relationships with parents, out of all sources of social support, helps to reduce children’s feelings of social intimidation by lowering their anxiety levels (Choi, 2014; Lee, 2020). Thus, even when YAMB perceive marginalization, maintaining close relationships with their parents may help reduce any intrapersonal obstacles (e.g., social withdrawal and self-doubt) in their career development, protecting them against adverse effects on their career maturity. Moreover, Choi and Yeon (2020) found that perceived parental attention to children’s interests and peer relationships reduced the social marginalization-related intimidation experienced by adolescents with migration backgrounds in South Korea. These studies support our finding that higher levels of intimacy with parents could mitigate perceived marginalization’s negative effects on individuals’ career maturity and mental health. However, the present study showed that outcomes varied somewhat based on participants’ immigration backgrounds, highlighting the need to better understand how the experiences of YAMB vary depending on whether they have immigrated to Korea or not.

The results of moderated-moderated mediation analysis showed that the mediation effect of career maturity moderated by intimacy with parents on the relationship between perceived marginalization and mental health was significant only for foreign-born YAMB. The correlation analysis between study variables indicated that foreign-born YAMB might perceive themselves as more marginalized than native-born YAMB. Also, the mediation effects of career maturity were only significant for foreign-born YAMB (see Figure 2B). Therefore, the effect of perceived marginalization on mental health through career maturity for foreign-born YAMB requires detailed discussion. First, this study’s finding that foreign-born YAMB experienced higher levels of perceived marginalization closely aligns with previous research pointing out the additional adaptation tasks and stressors (e.g., gaps in education years due to migration processes, difficulties in social adaptation, language barriers, the stigmas associated with their immigrant status, and bullying in media, neighborhoods, workplaces, and schools) that characterize abrupt or involuntary immigration processes, in addition to the shared stressors (e.g., racism, difficulties in interpersonal relationship, education or financial issues) that the native-born YAMB also experience (Brenick et al., 2012; Oh and Seo, 2012). Furthermore, a number of foreign-born YAMB aged 18 or older in South Korea reported concerns about career and economic problems and described often engaging in menial or repetitive labor such as drudgery due to their limited education levels, insufficient language abilities, and social prejudice (Bai, 2017). Participating in vocations that fall short of one’s expectations can trigger further marginalization as well as poverty, which can feed into a vicious cycle, making it more difficult for foreign-born YAMB to develop career maturity (Oh and Seo, 2012).

Similarly, Kim et al. (2012) reported that young immigrant adults often remain at lower levels of career awareness and experience fewer career preparation activities relative to their nonimmigrant counterparts. For example, career consciousness—the ability to properly perceive one’s value in life, occupation, principles, and career, and to make vocational decisions that suit and align with one’s identity (Korean Vocational Competency Development Institute, 1998)—is a necessary element in career decision making and career preparation. However, previous studies have found that a significant number of young immigrant adults’ experiences of discrimination and alienation prevent them from adjusting well to school environments, which makes it harder for them to properly form career consciousness from an early age (Kim, 2019, 2020b). Thus, they might struggle to develop concrete plans for their careers and experience lower levels of career maturity (Jo, 2011; Ryu et al., 2011; Yang, 2011; Ryu and Oh, 2012). Career immaturity during young adulthood—a period when it is generally necessary to initiate an active job search—is associated with increased mental health issues such as stress and depression (Park et al., 2008; Winderman et al., 2018). Moreover, young adults who migrate to South Korea are more likely to have specific expectations regarding professional achievement, and failure to meet those expectations in their early stages of settlement or experiencing career-related difficulties can trigger psychological problems such as frustration, anxiety, and escapism (Lee, 2004; Lee et al., 2008; Bai, 2017). In sum, these pieces of evidence suggest that the perceived marginalization experienced by foreign-born YAMB in young adulthood is likely to trigger mental health issues through reduced career maturity.

The moderated mediation effect that we found suggests that high intimacy with parents can mitigate the adverse effects of perceived marginalization experienced by foreign-born YAMB on mental health through career maturity. In other words, a positive relationship with parents can be a protective factor when migrating to another country and experiencing various adaptation conflicts. Furthermore, we found a negative association between perceived marginalization and mental health through career maturity among foreign-born YAMB with lower levels of intimacy with their parents. However, the mediating effect of career maturity was not significant for foreign-born YAMB with higher levels of intimacy with their parents.

Overall, many foreign-born YAMB experience complicated relationship dynamics and conflicts within their families, as well as emotional difficulties that can arise from witnessing their parents’ challenging acculturation processes (Lim, 2006; Oh, 2010; Yang et al., 2012; Uhm, 2013). Hence, during the immigration period, parent–child intimacy, perhaps stemming from effectively resolving family conflicts, may contribute to the re-establishment and maintenance of the newly formed family relationships, significantly contributing to the happiness, well-being, and competency young adults need to achieve independence and succeed in their careers (Lee and Kim, 2016; Go and Bai, 2017; Kim, 2020b). This finding aligns with previous studies showing that positive family experiences can buffer against the isolation and loneliness that arise during acculturation processes (Kim and Okazaki, 2014), and that trust in parents provides a sense of psychological stability that significantly helps individuals adapt (Kim, 2014, 2020b). In this respect, intimacy with parents carries more meaning for the foreign-born YAMB than for those born in Korea. Overall, our findings indicate that while foreign-born YAMB may experience low levels of career maturity as a result of their perceived marginalization, maintaining intimate relationships with their parents can protect against the deleterious influence of marginalization on career maturity, ultimately decreasing adverse mental health effects.

The study offers several important clinical implications. First and foremost, when working with young adults with migration backgrounds (YAMB) living in Korea, counselors should prioritize cultural sensitivity in understanding and addressing YAMB’s mental health issues. Given the study’s findings linking perceived marginalization to mental health through career maturity, counselors must be attuned to YAMB’s experiences of perceived marginalization. Counselors should create a safe and non-judgmental space for YAMB to discuss their feelings of social marginalization and alienation and help the client recognize how these experiences might be connected to their career challenges and mental well-being. Acknowledging and validating these experiences represents a critical first step that should not be overlooked in the therapeutic process with this population. This is especially important because when YAMB perceive counselors as members of the mainstream society that has marginalized them, expressing their feelings related to marginalization and discrimination can be extremely challenging. Failure to address this issue can hinder progress in counseling.

Second, counselors should be cognizant of the differing experiences and needs of foreign-born YAMB compared to those born in Korea. Tailoring therapeutic interventions to address the unique challenges faced by foreign-born YAMB is essential. Counselors should take proactive approach to mitigate the negative effects of perceived marginalization by facilitating career maturity enhancement and improved intimacy with parents among foreign-born YAMB. These individuals may encounter limitations in understanding the job-seeking process in Korea, as well as accessing job-related information and career role models due to their narrower social networks compared to Korean-born YAMB. Therefore, counselors should adopt a social justice-based career counseling approach and extend support beyond the therapy room (Rhie et al., 2018). This can be achieved by connecting foreign-born YAMB with external resources, such as skill-specific workshops, community job fairs, job-related education programs, and even language programs if necessary.

Additionally, to help foreign-born YAMB improve intimacy with their parents, therapists should make efforts to acquire a deep understanding of the cultural context, values, and communication styles of both the YAMB and their parents, as these factors and related differences can significantly influence their level of intimacy during the acculturation process. This heightened awareness will enable therapists to approach family dynamics with cultural sensitivity, avoiding misunderstandings and stereotypes. Counselors can also share this awareness with clients and assist them in fostering more open and effective communication with their parents to resolve conflicts that may strain their relationship. Strengthening the bond with their parents can serve as a buffer against experiences of marginalization, provide emotional support amid career difficulties, and contribute to improved mental health.

This study contributed the literature in the following aspects. First, we focused on perceived marginalization, capturing subjective feelings of isolation that go beyond assessments of socioeconomic status or income level. While most studies of multicultural families have focused on experiences of discrimination, minorities tend to experience more covert and the micro forms of oppression in Korea. Thus, studies examining overt discrimination cannot thoroughly reflect the challenges underrepresented populations face (Pascoe and Richman, 2009). Therefore, by examining the subjective perspectives of young adults from migrant backgrounds (YAMB) and analyzing how these perspectives relate to career issues and overall mental health, our study contributes meaningfully to the literature.

Second, in contrast to previous studies focusing on children, adolescents, and married female immigrants in Korea, our study sheds light on the experiences of YAMB during the developmental phase in which they become independent from their original families and start their roles as adults. Furthermore, we found that the marginalization perceived by YAMB negatively affects their mental health through career maturity. This finding implies that Korea as a society still discriminates against immigrants and fails to provide adequate social structures to support the psychological adaptation of YAMB—who are at prime employment age. To become a multicultural society that fully embraces YAMB in the era of rapid globalization (Yoo and Kim, 2015), Korea needs to foster an intentional multicultural orientation in the job market that promotes culturally sensitive policies to help young adults with migrant backgrounds address their career issues.

Third, as pointed out by Lee and Kim (2016), the paucity of research exploring differences between native and foreign-born YAMB in Korea highlights the need to carefully explore and understand the unique experiences and challenges of immigrants by nativity status; such an effort will facilitate the development of specific interventions. Our study provides initial evidence of some key differences between native and foreign-born YAMB. As such, tailored support systems and policies to meet the unique needs of YAMB based on their nativity status should be developed.

Despite its significant contributions, this study has several limitations. First, although we identified perceived marginalization and intimacy with parents as important factors in the psychosocial adaptation of young adults from multicultural families, relatively few previous studies have explored these concepts with YAMB, and more valid scales should be developed—particularly to assess marginalization. Indeed, further research is needed to capture the nuanced subjective feelings of marginalization that accompany discriminatory experiences and to examine the specific effects of intimacy with parents as a protective factor against perceived marginalization. In particular, keeping in mind that the effects of perceived marginalization can differ depending on the socio-cultural context, researchers should develop a Korean version of the perceived marginalization scale to reflect the experiences of young Korean adults with migration backgrounds. Also, more research is needed to develop culturally responsive psychological interventions for foreign-born YAMB so that they can receive assistance in better coping with various adaptation issues while moving into a new culture; for example, Kim (2020a) emphasized the need for early counseling interventions to support the adaptation of young adults from multicultural families. Similarly, support systems to help foreign-born YAMB resolve their career or mental health issues, particularly when they lack intimate parental support to alleviate the negative effects of perceived marginalization, are sorely needed. For example, YAMB above 25 in Korea miss out on government support because the Multicultural Family Support Act limits government assistance to migrant young adults under 24 (Song et al., 2015). Therefore, various internal and external resources such as appropriate counseling, mentoring programs, and social support systems need to be further developed through future research, government guidelines, and policies, which in turn can help decrease the negative effects of perceived marginalization on immigrant young adults’ mental well-being.

Second, while we made a meaningful attempt to consider the various demographic characteristics of participants’ backgrounds, we were unable to include certain potentially important demographic variables, such as language preference, ethnicity, and race; as such, we did not examine differences based on these characteristics within the single group of YAMB. YAMB’s experiences within Korea such as marginalization, microaggressions, and overt discrimination can vary considerably depending on certain specific demographic characteristics, which in turn can affect mental health differently. Thus, studies that intentionally examine within-group differences should be conducted with YAMB to better understand the unique stressors experienced by subgroups and create nuanced intervention and prevention programs for YAMB as needed.

## Data availability statement

The raw data supporting the conclusions of this article will be made available by the authors, without undue reservation.

## Ethics statement

The studies involving humans were approved by Institutional Review Board of Dankook University in South Korea. The studies were conducted in accordance with the local legislation and institutional requirements. The participants provided their written informed consent to participate in this study.

## Author contributions

SJ conceptualized and designed the study, oversaw data collection, and contributed significantly to manuscript writing. JR conducted data analysis using appropriate statistical methods and interpreted the results. She also played a significant role in manuscript writing, particularly in the sections related to data analysis and result interpretation. KY provided the overall supervision, overseeing and providing guidance throughout the research process. He also actively participated in writing the methodology, data analysis, and the result interpretation. PK conducted an extensive literature review and significantly contributed to the writing of the discussion and the conclusion part of the manuscript, synthesizing existing knowledge and contributing to the writing of the overall manuscript. MK actively contributed to the data collection as well as the manuscript reviewing processes. All authors contributed to the article and approved the submitted version.
